# Differential Impact of Calcitriol and Its Analogs on Tumor Stroma in Young and Aged Ovariectomized Mice Bearing 4T1 Mammary Gland Cancer

**DOI:** 10.3390/ijms21176359

**Published:** 2020-09-02

**Authors:** Artur Anisiewicz, Agata Pawlik, Beata Filip-Psurska, Joanna Wietrzyk

**Affiliations:** Department of Experimental Oncology, Hirszfeld Institute of Immunology and Experimental Therapy, Polish Academy of Sciences, 53-114 Wroclaw, Poland; agata.maria.pawlik@gmail.com (A.P.); beata.filip-psurska@hirszfeld.pl (B.F.-P.); joanna.wietrzyk@hirszfeld.pl (J.W.)

**Keywords:** breast cancer, tumor stroma, metastasis, immunity, elderly, calcitriol, vitamin D

## Abstract

(1) Background: Vitamin D compounds (VDC) are extensively studied in the field of anticancer properties, including breast cancer. Previously, we showed that calcitriol and its analogs (PRI-2191 and PRI-2205) stimulate metastasis in 4T1 murine mammary gland cancer models in young mice, whereas the reverse effect was observed in aged ovariectomized (OVX) mice; (2) Methods: We determined the phenotype of monocytes/macrophages using FACS and examined the expression of selected genes and proteins by Real-Time PCR and ELISA; (3) Results: Activities of VDC are accompanied by an increase in the percentage of Ly6C^low^ anti-inflammatory monocytes in the spleen of young and a decrease in aged OVX mice. Treatment of young mice with VDC resulted in an increase of CCL2 plasma and tumor concentration and Arg1 in tumor. In later stage of tumor progression the expression of genes related to metastasis in lung tissue was decreased or increased, in old OVX or young mice, respectively; (4) Conclusions: Pro- or anti-metastatic effects of calcitriol and its analogs in young or aged OVX mice, respectively, can be attributed to the differences in the effects of VDC on the tumor microenvironment, as a consequence of differences in the immunity status of young and aged mice.

## 1. Introduction

Numerous studies conducted in recent years have indicated that interactions between cancer cells and their microenvironment play a significant role at each stage of tumor progression [1]. Cancer cells recruit stromal, vascular, and immunological cells to their environment through secreted cytokines, chemokines, and growth factors, which further results in the formation of tumor stroma [2]. Antitumor activity of the immune system is often observed in the initial stage of tumor development and is associated with the action of natural killer cells, M1-like tumor-associated macrophages (TAMs), N1 neutrophils, TCD8 cytotoxic cells, and Th1 lymphocytes. However, due to the persistence of pathological inflammation and various factors released by cancer cells, immunosuppression of the tumor microenvironment (TME) is often observed, which is manifested by the abundant presence of tumor-promoting cancer-associated fibroblasts, M2-like TAMs, N2 neutrophils, mast cells, myeloid-derived suppressor cells, regulatory T cells (Tregs), and Th2 lymphocytes (reviewed at: [3]).

The anticancer properties of calcitriol, a biologically active form of vitamin D, are widely discussed, and include antiproliferative and anti-inflammatory effects, induction of apoptosis, stimulation of differentiation, and inhibition of angiogenesis, invasion, and metastasis (for details see references mentioned in [4]). In vitro studies indicate that calcitriol and its analogs efficiently inhibit the proliferation of human and mice breast cancer cell lines associated with a less invasive phenotype [5,6,7,8,9], while more aggressive models of breast cancer show limited sensitivity to vitamin D compounds or are found to be sensitive only at high doses [6,8,9,10]. Moreover, animal studies have shown that vitamin D supplementation in mouse feed or calcitriol administration inhibited tumor growth or metastasis in mice with human breast cancer xenograft [11,12], in allogenic transplant models [13,14], as well as in the MMTV-PyMT spontaneous mouse mammary gland cancer [15]. In parallel, a deficiency of vitamin D in the diet accelerated the rate of tumor growth [14,15], and vitamin D receptor (VDR) ablation further enhanced the tumor volume and metastatic potential [16,17]. On the other hand, our research group recently published a study in which we showed stimulation of metastasis after administration of calcitriol and its analogs in the 4T1 mouse model of breast cancer [9], while Cao et al. reported a significant increase in tumor volume after vitamin D treatment [18]. The results of observational studies and randomized clinical trials (RCTs) are not conclusive and indicate a lack or only small benefits of vitamin D supplementation in breast cancer pathogenesis [19,20]. However, it is also postulated that vitamin D deficiency may promote the development of breast cancer and is usually associated with a poor prognosis [21,22].

An important role of vitamin D is its impact on the immune system, both innate and adaptive [23]. The active form of vitamin D inhibits the differentiation of naive T cells to Th17 cells [24], while stimulation of the Treg population is increased [24]. Furthermore, calcitriol shifts the balance of Th lymphocytes toward Th2 cells [25] and inhibits the proliferation of B lymphocytes and production of immunoglobulins by them [26]. Moreover, vitamin D enhances the antibacterial properties of monocytes/macrophages [27]; however, on the other hand, it contributes to reduced secretion of pro-inflammatory cytokines (interleukin IL-1, IL-6, IL-23, tumor necrosis factor alpha (TNF-α)) [28,29] and decreased ability to present antigens due to lowered expression of CD40, CD80, CD86, and MHC class II molecules [30]. It has also been demonstrated that vitamin D stimulated the secretion of immunosuppressive IL-10 by peripheral blood mononuclear cells (PBMCs) infected with *Mycobacterium tuberculosis* [28], whereas calcitriol-stimulated monocytes showed reduced expression of *IL-12* gene encoding a key cytokine responsible for inducing Th1-type immune response and macrophage polarity for the M1 class [31]. To date, it has not been described whether vitamin D affects the polarization of the TAM in the tumor. However, Yin et al. proved that THP-1 monocytes showed increased expression of markers characteristic of the M2 class following calcitriol treatment [32]. Similarly, RAW 264.7 and U937 macrophages stimulated to M1 class were repolarized to M2 class after incubation with vitamin D, which was manifested by decreased production of IL-12, IL-6, inducible nitric oxide synthase (iNOS), and TNF-α and increased expression of CD206, IL-10, and Arg1 [33].

In our previous studies, we have demonstrated that calcitriol and its analogs, PRI-2191 and PRI-2205, increased the number of metastases in the lungs of 6–8-week-old mice bearing 4T1 mammary gland cancer cells, without affecting the growth of the primary tumor. This effect was associated, among others, with increased osteopontin (OPN) level, improved tumor blood perfusion [9] and prevalence of Th2 response, elevated activity of Treg cells [34], and increased differentiation of Th17 cells [35]. However, these changes were not noticeable in aged 60-week-old ovariectomized (OVX) mice, where the transient anti-metastatic effect was visible in mice treated with vitamin D compounds [35,36]. Additionally, we noted a decrease in the percentage of TCD4+, TCD8+, Treg, and NK cells for splenocytes of young mice [34]. On the other hand, for OVX 60-week-old mice, the reduction in the TCD4+, TCD8+, and Treg populations was also visible, but no significant differences were noted for the percentage of NK cells [35]. Therefore, we decided to extend our research that we carried out on the tissues collected in previous experiments. Herein, we present in vivo and in vitro results aimed at broadening the available knowledge about the influence of vitamin D and its analogs on the tumor microenvironment in the 4T1 mouse mammary gland cancer model depending on the age of the mouse, young or aged OVX.

## 2. Results

### 2.1. Calcitriol and Its Analogs Reduced Whole Blood Monocytes in Aged OVX Mice in the Late Stage of Tumor Progression

**Figure 1:**
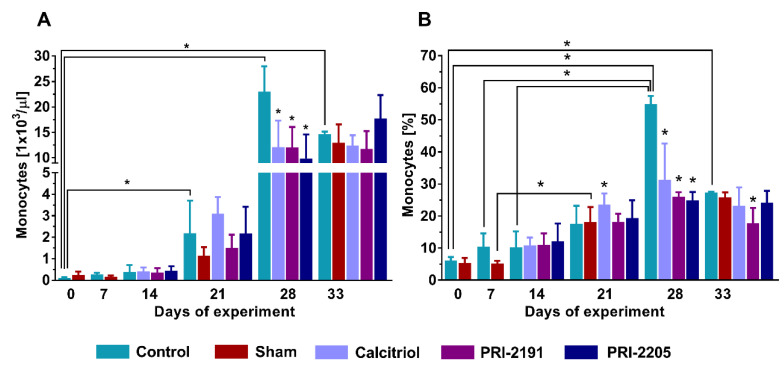
Whole blood monocytes measurement in 4T1 tumor-bearing aged OVX mice. (**A**) Total number of monocytes in whole blood. (**B**) Percentage of monocytes in whole blood among all leukocytes. Briefly, 60-week-old mice were subjected to ovariectomy or sham surgery. After 4 weeks, 4T1 cells were inoculated (day 0) orthotopically and subcutaneous administration of the tested compounds (three times a week) was initiated on day 7 at the following doses: calcitriol, 0.5 µg/kg; PRI-2191, 1.0 µg/kg; and PRI-2205, 10.0 µg/kg. The number of mice analyzed was 3–7 per group. Data presentation: (**A**,**B**), mean with standard deviation (SD). Statistical analysis: (**A**,**B**), Dunn’s multiple comparison test. * *p* < 0.05 as compared to control animals on the relevant day of treatment or as indicated.

The increasing effect of calcitriol and its analogs on the percentage of monocytes in the whole blood of young mice bearing 4T1 tumor is presented in our previous work [34]. In the case of control old OVX mice, we observed a systematic increase in the number of monocytes during tumor progression, both in the total amount of monocytes (Figure 1A) and the percentage of monocytes among all leukocytes (Figure 1B). In the early stage of tumor progression, no effects of vitamin D compounds were observed, with the exception of a statistically significant increase (*p* < 0.05) in the percentage of monocytes in calcitriol-treated mice on day 21 (Figure 1B). However, in the late stage of tumor progression (day 28), we observed a significant decrease (*p* < 0.05) in the treated mice, both in quantity (Figure 1A) and percentage (Figure 1B) of monocytes. On day 33, the effects related to vitamin D compounds were not observed, except for a reduction in the monocyte percentage following treatment with PRI-2191. There were no significant differences between OVX and sham mice throughout the duration of the experiment (Figure 1A,B).

### 2.2. Calcitriol and Its Analogs Increase or Decrease the Ly6C^low^ to Ly6C^high^ Ratio of Splenic Monocytes in Young and Aged OVX Mice, Respectively, in the Late Stage of Cancer Progression

**Figure 2:**
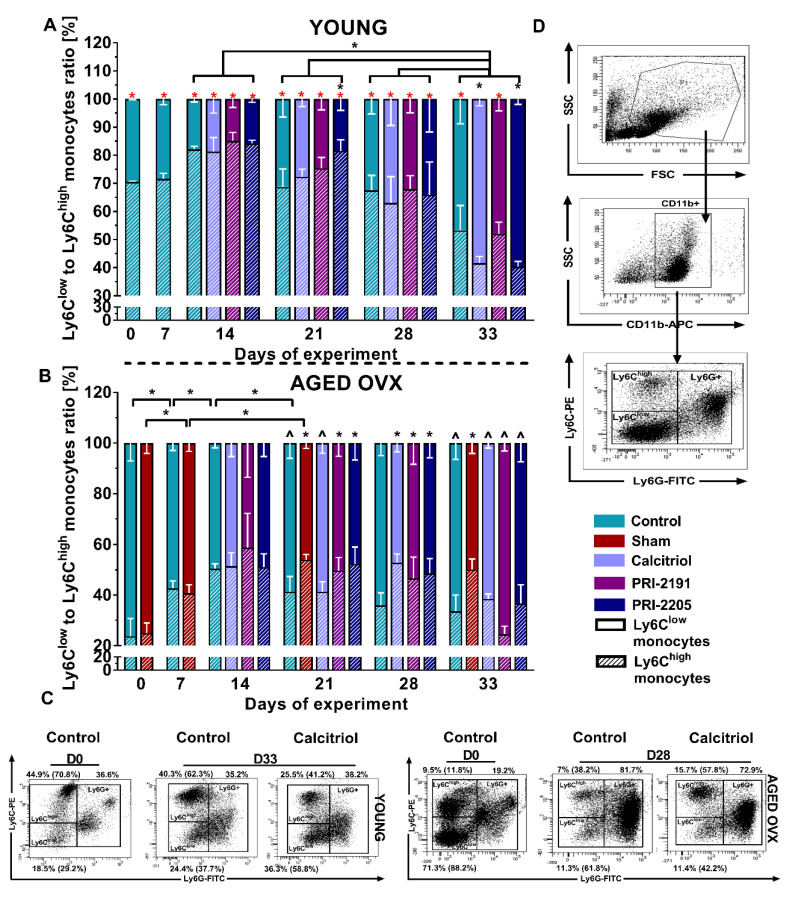
The Ly6C^low^ to Ly6C^high^ ratio of splenic monocytes in 4T1 tumor-bearing young and aged OVX mice. Ly6C^low^ to Ly6C^high^ ratio of splenic monocytes in (**A**) young or (**B**) aged OVX mice is presented. (**C**) Representative dot plots of selected analysis with the % of cells of each gate performed on days 0 and 28, and 33. The values in parentheses represent the percentage of monocytes of individual classes among all monocytes. (**D**) Gating strategy used in the analysis. 1 × 10^6^ viable cells were used for cytometric analysis. Firstly, monocytes were gated as CD11b-positive and Ly6G-negative cells. Then, the monocytes were differentiated due to the expression of the Ly6C marker. Briefly, 60-week-old mice were subjected to ovariectomy or sham surgery. After 4 weeks, 4T1 cells were inoculated (day 0) orthotopically into aged OVX mice or 6–8-week-old young mice, and subcutaneous administration of the tested compounds (three times a week) was initiated on day 7 at the following doses: calcitriol, 0.5 µg/kg; PRI-2191, 1.0 µg/kg; and PRI-2205, 10.0 µg/kg. The number of mice analyzed was 3–7 per group. Data presentation: (**A**,**B**), mean with standard deviation (SD). Statistical analysis: (**A**) Dunn’s multiple comparison test and (**B**) Dunnett’s multiple comparison test. * *p* < 0.05 as compared to control animals or ^ *p* < 0.05 as compared do control sham animals on the relevant day of treatment or * *p* < 0.05 as compared to corresponding group of aged OVX mice or as indicated.

For healthy young mice (day 0), patrolling monocytes Ly6C^low^ accounted for nearly 30% of the total monocytes (Figure 2A), whereas in old OVX mice, the percentage of this population was about 75% (Figure 2B). On days 7 and 14 after 4T1 cell transplantation, the percentage of Ly6C^low^ monocytes decreased with a simultaneous increase in a subpopulation of inflammatory monocytes Ly6C^high^, reaching 18% and 50% for the control group on day 14, respectively, in young and aged OVX mice (Figure 2A,B). In the case of young mice, a significant increase (*p* < 0.05) in Ly6C^low^ monocytes was observed in the following days (Figure 2A). In the early stage of tumor progression, no significant effect of the therapy was noticed, with the exception of a decrease in the Ly6C^low^ population on day 21 after treatment with PRI-2205 (*p* < 0.05, Figure 2A). However, in the late phase of cancer progression, a significant increase (*p* < 0.05) in Ly6C^low^ monocytes was observed on day 33 in mice treated with calcitriol and PRI-2205 (Figure 2A). Different observations were noted for aged OVX mice. On day 21, both analogs lowered the percentage of Ly6C^low^ monocytes, while on day 28, an increase in the percentage of this subpopulation was seen in comparison to the control group in all the study groups (*p* < 0.05, Figure 2A).

Moreover, young mice on days D0 to D28 in all groups had a significantly higher percentage of Ly6C^high^ monocytes (*p* < 0.05, Figure 2A) compared to aged OVX mice. During tumor progression, the percentage of Ly6C^low^ monocytes increased in young mice, while in aged OVX mice was at a similar level from the 14th day of the experiment. On day 33, young and old OVX mice generally had a similar Ly6C^low^:Ly6C^high^ ratio (significant differences were noted only for the control and PRI-2191 treated groups).

### 2.3. Expression of Genes Related to Invasion and Metastasis in the Lung Tissue of 4T1 Tumor-Bearing Young and Aged OVX Mice

Herein, we present the results of the expression of the following genes related to invasion and metastasis: *Csf1* (colony-stimulating factor 1), *Drg1* (developmentally regulated GTP binding protein), *Flt1* (Fms-related tyrosine kinase 1), *Mmp14* (matrix metalloproteinase-14), *Nedd9* (neural precursor cell expressed, developmentally downregulated 9), *Nf2* (neurofibromin 2), *Plaur* (plasminogen activator urokinase receptor), *Spp1* (secreted phosphoprotein 1, osteopontin), and *Tgfb1* (transforming growth factor beta 1), selected on the basis of gene screening described by us in [9,34] and genes related to inflammation: *Ccr6* (chemokine receptor 6), *Cxcr4* (C-X-C chemokine receptor type 4) and *Foxo1* (Forkhead box protein O1). For young mice, the results for the expression of *Spp1* and *Tgfb1* genes are published in [34].

**Figure 3:**
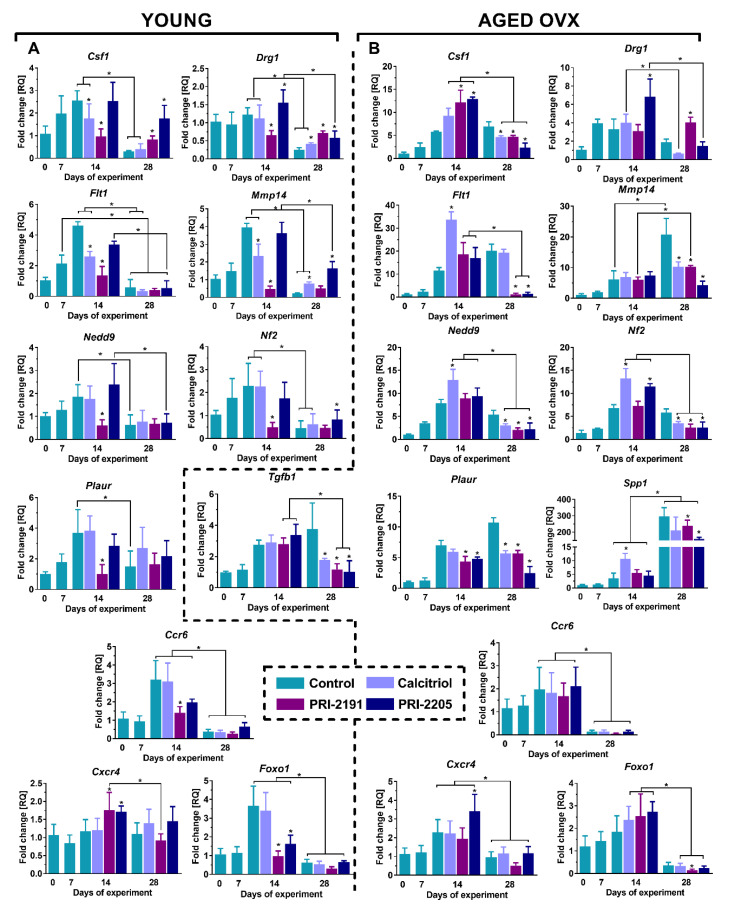
Changes related to gene expression in the lungs of 4T1 tumor-bearing young and aged OVX mice. Expression of selected genes in lung tissue of (**A**) young or (**B**) aged OVX mice estimated by real-time PCR is presented. The analysis was performed on tissue samples harvested at days 0, 7, 14, and 28. 25 ng of cDNA was used for a single reaction and each sample was applied in triplicate (day 0). Ribosomal protein L13a gene (*Rpl13a*) was used as a housekeeping gene in the analysis. Briefly, 60-week-old mice were subjected to ovariectomy or sham surgery. After 4 weeks, 4T1 cells were inoculated (day 0) orthotopically into aged OVX mice or 6–8-week-old young mice, and subcutaneous administration of the tested compounds (three times a week) was initiated on day 7 at the following doses: calcitriol, 0.5 µg/kg; PRI-2191, 1.0 µg/kg; and PRI-2205, 10.0 µg/kg. The number of mice analyzed was 3–7 per group. Data presentation: (**A**,**B**), mean with standard deviation (SD). Statistical analysis: (**A**,**B**), Dunn’s multiple comparison test. * *p* < 0.05 as compared to control animals on the relevant day of treatment or as indicated.

By day 14, there was an increase in the expression of the investigated genes in the group of control young mice; however, in the late phase of tumor progression (day 28), a decrease in the level of gene transcription was observed in the untreated mice mostly (Figure 3A). On day 14, PRI-2191 was found to downregulate the expression of all the examined genes. Calcitriol also acts in a similar fashion, by lowering the expression of *Csf1*, *Flt1*, and *Mmp14* genes (*p* < 0.05, Figure 3A). Except for the expression of *Drg1*, which was found to be significantly increased, there were no differences between control and PRI-2205-treated mice with regard to the expression of other genes on that day. On day 28, both analogs significantly upregulated the mRNA level of *Csf1*, and PRI-2205 elevated *Nf2* transcription level (*p* < 0.05, Figure 3A). Moreover, all the tested compounds increased *Drg1* gene expression, while calcitriol and PRI-2205 also upregulated the mRNA level of *Mmp14* (*p* < 0.05, Figure 3A). In the case of inflammatory markers, on day 14 there was an increase in the expression of *Ccr6* and *Foxo1* mRNA, especially in the control group and the group treated with calcitriol (relative to day 0 and 7 of the experiment), after which on day 28 the expression of the studied genes decreased significantly (Figure 3A, *p* < 0.05) in all groups below the level observed for healthy lungs (day 0). Otherwise, expression of *Cxcr4* increased on day 14 in the PRI-2191 and PRI-2205 groups, however, this effect was not observed on day 28 (expression at the level noted for normal lungs).

Different observations were noted for aged OVX mice; namely, during tumor progression, an increase in the expression of examined genes was generally observed (Figure 3B). On day 14 of the experiment, calcitriol increased the mRNA levels of *Flt1*, *Nedd9*, *Spp1*, and *Nf2* (*p* < 0.05, Figure 3B). Moreover, expression of *Nf2* was also elevated in the PRI-2205–treated groups, and a similar trend was observed for *Drg1*. Both analogs were found to upregulate *Csf1* and downregulate *Plaur* gene expression (*p* < 0.05, Figure 3B). In the late stage of tumor progression (day 28), there was a reduction in the levels of *Csf1*, *Mmp14*, *Nedd9*, *Nf2*, *Plaur*, and *Tgfb1* genes by all the investigated compounds. Furthermore, both analogs decreased *Flt1* and *Spp1* mRNA expression, while upregulation was noted only for *Drg1* after PRI-2191 treatment (*p* < 0.05, Figure 3B). Analogously to the young mice, the expression of *Ccr6* and *Foxo1* for aged OVX mice was increased in all groups on day 14 and then decreased below the level observed in normal mice (D0). For *Cxcr4*, an increase in expression was noted on day 14 in all groups, significant for the PRI-2205 treated group as compared to control mice (*p* < 0.05). However, on day 28, expression of *Cxcr4* in all groups was comparable to that noted in normal lungs (Figure 3B).

### 2.4. Expression of Genes Related to Invasion and Metastasis in the Tumor Tissue of 4T1 Tumor-Bearing Aged OVX Mice

Herein, we present the results of the expression of the following genes related to invasion and metastasis: *Csf1, Drg1, Flt1, Mmp13, Mmp14, Nedd9, Nf2, Plaur, Spp1*, and *Tgfb1* selected on the basis of gene screening described by us in [9,34]. For young mice, the results are published in [9].

**Figure 4:**
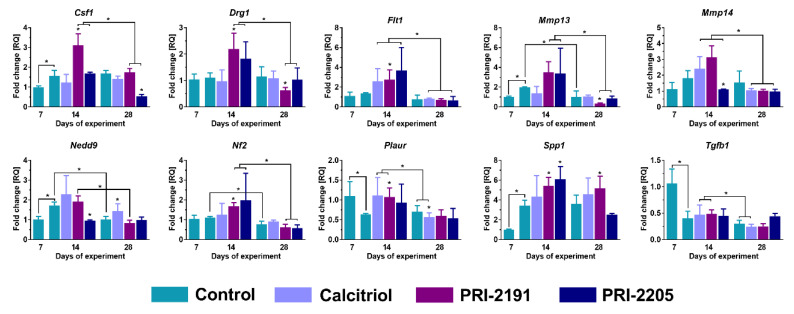
Changes related to gene expression in tumor tissue of 4T1 tumor-bearing aged OVX mice. Expression of selected genes in tumor tissue of aged OVX mice estimated by real-time PCR. The analysis was performed on tissue samples harvested at days 7, 14, and 28. 25 ng of cDNA was used for a single reaction and each sample was applied in triplicate (day 0). Phosphoglycerate Kinase 1 (*Pgk1*) was used in the analysis as a housekeeping gene. Briefly, 60-week-old mice were subjected to ovariectomy or sham surgery. After 4 weeks, 4T1 cells were inoculated (day 0) orthotopically and the subcutaneous administration of tested compounds (three times a week) was initiated on day 7 at the following doses: calcitriol, 0.5 µg/kg; PRI-2191, 1.0 µg/kg; and PRI-2205, 10.0 µg/kg. The number of mice analyzed was 3–7 per group. Data presentation: mean with standard deviation (SD). Statistical analysis: Dunn’s multiple comparison test. * *p* < 0.05 as compared to control animals on the relevant day of treatment or as indicated.

In general, the expression of the examined genes on day 14 was insensibly higher or comparable to that observed on day 7, and there was only a slight decrease or no change at all on day 28 (Figure 4). There was no significant effect of calcitriol on mRNA expression of the tested genes on day 14. Similar results were noted for PRI-2205 on this day, with the exception of downregulated effect on mRNA levels of *Mmp14* and *Nedd9* and upregulated impact on the level of Spp1 (*p* < 0.05, Figure 4). PRI-2191 upregulated the mRNA expression of most of the genes (*Csf1, Drg1, Flt1, Nf2, Plaur*, and *Spp1*, *p* < 0.05, Figure 4). There was no effect of the used treatment on the expression of *Mmp13* and *Tgfb1* genes.

On day 28, no effects on the expression of *Flt1, Mmp14, Nf2*, and *Tgfb1* genes were observed in the treatment groups. On this day, calcitriol increased the mRNA level of *Nedd9*, while *Plaur* level was decreased (*p* < 0.05, Figure 4). PRI-2205 reduced the expression of *Csf1* and PRI-2191 lowered the expression of *Drg1* and *Mmp13* genes. On the other hand, mRNA level of *Spp1* was found to be upregulated following PRI-2191 treatment (*p* < 0.05, Figure 4).

### 2.5. Differential Effect of Vitamin D Compounds on the Expression of Proteins Associated with Tumor Microenvironment in 4T1 Tumor-Bearing Young and Aged OVX Mice

Due to the increase in the number of metastases in the lungs of young mice treated with vitamin D compounds [9], we decided to estimate the effect of calcitriol and its analogs on the level of proteins expressed by the tumor microenvironment cells, especially macrophages in tumor tissue.

**Figure 5:**
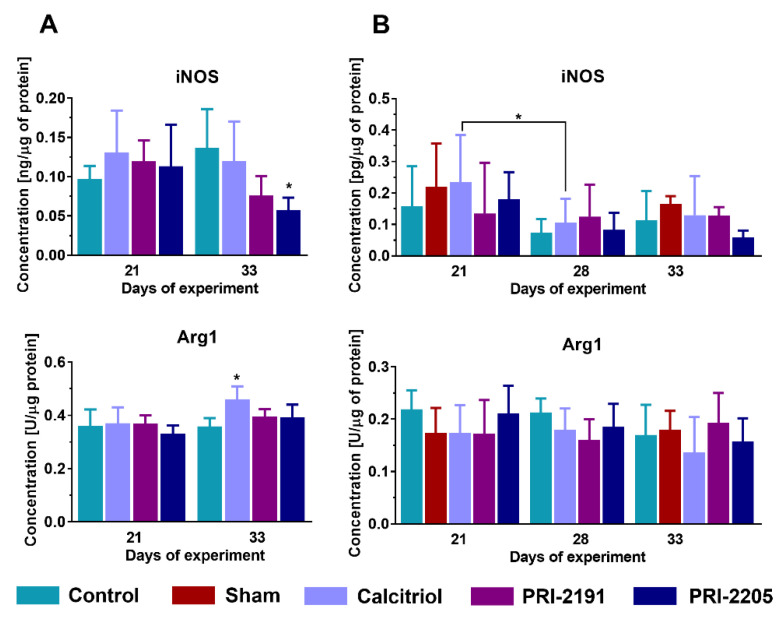
Changes related to protein expression in tumor tissue of 4T1 tumor-bearing (**A**) young and (**B**) aged OVX mice. Expression of selected proteins in tumor tissue measured using ELISA assays. The analysis was performed on samples harvested at days 14 and 28 (young mice) or days 21, 28, and 33 (aged OVX). Briefly, 60-week-old mice were subjected to ovariectomy or sham surgery. After 4 weeks, 4T1 cells were inoculated (day 0) orthotopically into aged OVX mice or 6–8-week-old young mice, and subcutaneous administration of tested compounds (three times a week) was initiated on day 7 at the following doses: calcitriol, 0.5 µg/kg; PRI-2191, 1.0 µg/kg; and PRI-2205, 10.0 µg/kg. The number of mice analyzed was 3–7 per group. Data presentation: (**A**), mean with standard deviation (SD). Statistical analysis: (**A**), Kruskal–Wallis multiple comparison test. * *p* < 0.05 as compared to control animals on the relevant day of treatment or as indicated. iNOS—inducible nitric oxide synthase; Arg1—arginase 1.

It has been reported that calcitriol elevated the level of Arg1 in tumor tissue of young mice, whereas PRI-2205 lowered iNOS level at day 33 (*p* < 0.05, Figure 5A), and a similar trend was observed for PRI-2191 (*p* = 0.0806). There were no significant differences between the groups in aged OVX mice (Figure 5B). However, the opposite trend was seen to that observed for young mice—calcitriol and PRI-2191 slightly increased iNOS expression on day 28, while Arg1 expression was decreased on day 21 of the experiment after treatment with calcitriol and PRI-2191 (*p* = 0.097 and *p* = 0.0843, respectively) and on day 28 of the experiment also after calcitriol (*p* = 0.1210) and PRI-2191 (*p* = 0.0620).

### 2.6. Calcitriol and Its Analogs Elevate the Level of CCL2 Chemokine in the Late Stage of Cancer Progression in 4T1 Tumor-Bearing Young Mice

**Figure 6:**
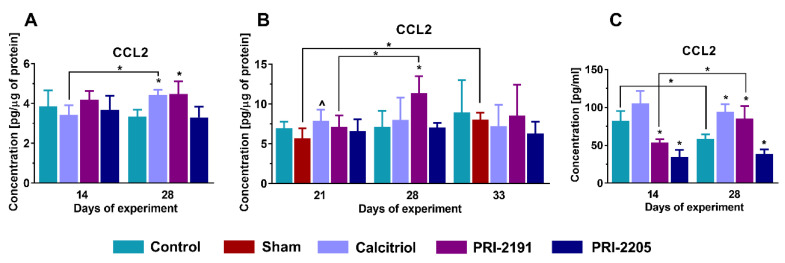
Changes related to CCL2 expression in 4T1 tumor-bearing young and aged OVX mice. Expression of CCL2 in (**A**,**B**) tumor tissue or (**C**) plasma of (**A**,**C**) young or (**B**) aged OVX mice measured using ELISA assays. The analysis was performed on samples harvested at days 14 and 28 (young mice) or days 21, 28, and 33 (aged OVX). Briefly, 60-week-old mice were subjected to ovariectomy or sham surgery. After 4 weeks, 4T1 cells were inoculated (day 0) orthotopically into aged OVX mice or 6–8-week-old young mice, and subcutaneous administration of tested compounds (three times a week) was initiated on day 7 at the following doses: calcitriol, 0.5 µg/kg; PRI-2191, 1.0 µg/kg; and PRI-2205, 10.0 µg/kg. The number of mice analyzed was 3–7 per group. Data presentation: (**A**–**C**), mean with standard deviation (SD). Statistical analysis: (**A**–**C**), Kruskal–Wallis multiple comparison test. * *p* < 0.05 as compared to control animals or ^ *p* < 0.05 as compared do control sham animals on the relevant day of treatment or as indicated. CCL2—chemokine (C-C motif) ligand 2.

Calcitriol and PRI-2191 significantly increased (*p* < 0.05) the concentration of chemokine CCL2 in the tumor tissue of young mice in the late stage of cancer progression (Figure 6A), while this effect was not observed in aged OVX mice, with the exception of PRI-2191 (*p* < 0.05) which showed an increasing impact on day 28 (Figure 6B). Due to the limited amount of sample available for analysis, plasma CCL2 concentration was measured only in young mice. Both analogs lowered CCL2 levels in plasma at day 14 (*p* < 0.05, Figure 6C). On day 28, this effect was maintained for the PRI-2205 analog, while calcitriol and PRI-2191 significantly increased the level of CCL2 (*p* < 0.05, Figure 6C).

### 2.7. Effect of Calcitriol and Its Analogs on Cytokine Expression in 4T1 Tumor-Bearing Aged OVX Mice

In this study, we present the results for the expression of TGF-β, IL-10, and IL-1β, obtained from tissues harvested from aged OVX mice. For young mice, the results have been included in our previous works [9,34].

**Figure 7:**
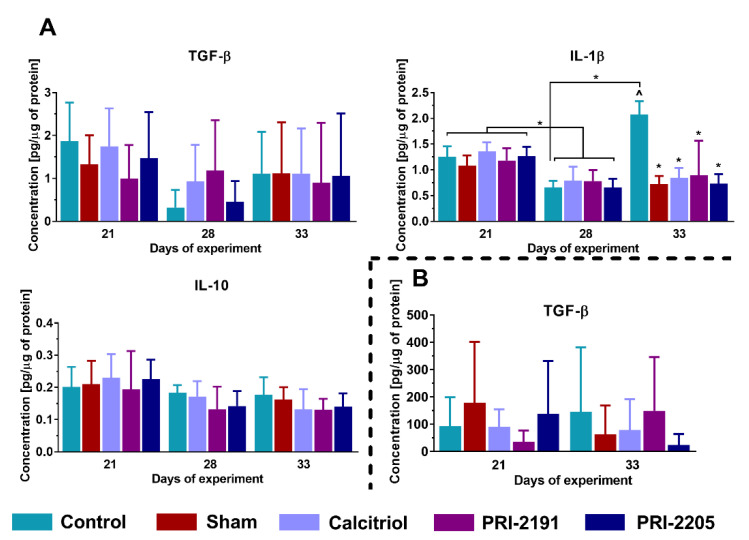
Changes related to protein expression in 4T1 tumor-bearing aged OVX mice. Expression of selected proteins in (**A**) tumor tissue or (**B**) plasma of aged OVX mice measured using ELISA assays. The analysis was performed on samples harvested at days 21, 28, and 33. Briefly, 60-week-old mice were subjected to ovariectomy or sham surgery. After 4 weeks, 4T1 cells were inoculated (day 0) orthotopically and the subcutaneous administration of tested compounds (three times a week) was initiated on day 7 at the following doses: calcitriol, 0.5 µg/kg; PRI-2191, 1.0 µg/kg; and PRI-2205, 10.0 µg/kg. The number of mice analyzed was 3–7 per group. Data presentation: mean with standard deviation (SD). Statistical analysis: Kruskal–Wallis multiple comparison test. * *p* < 0.05 as compared to control animals or ^ *p* < 0.05 as compared do control sham animals on the relevant day of treatment or as indicated. IL-1β—interleukin 1β; IL-10—interleukin 10; TGF-β—transforming growth factor β.

Vitamin D compounds did not affect the concentration of TGF-β and IL-10 in the tumor (Figure 7A) and TGF-β in the plasma (Figure 7B) tissues. However, PRI-2191 showed a tendency to lower the level of IL-10 on day 28 (*p* = 0.0563) compared to control mice. In the case of IL-1β, all the tested agents decreased the level of this cytokine on day 33 of the experiment (*p* < 0.05, Figure 7A).

### 2.8. Diversified Effect of Vitamin D Compounds on CCL2 and OPN Secretion by Murine Fibroblasts (BALB/3T3) and Macrophages (RAW 264.7)

**Figure 8:**
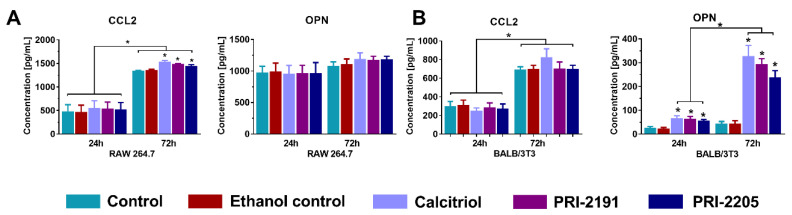
Expression of CCL2 and OPN in BALB/3T3 and RAW 264.7 cell cultures. Secretion of CCL2 and OPN into supernatants by (**A**) RAW 264.7 and (**B**) BALB/3T3 cells measured using ELISA assays. Briefly, 3 × 10^5^ cells were seeded on Tissue Cultured dishes. The following day, vitamin D compounds at 100 nM concentration were added and incubated for 24 or 72 h, and four independent repetitions were performed. Cell culture supernatants for ELISA assays were collected after 24 and 72 h. Data presentation: (**A**,**B**), mean with standard deviation (SD). Statistical analysis: (**A**,**B**), Sidak’s multiple comparison test. * *p* < 0.05 as compared to both controls or as indicated. CCL2—chemokine (C-C motif) ligand 2; OPN—osteopontin.

CCL2 and OPN levels were measured in RAW 264.7 (Figure 8A) macrophage and BALB/3T3 (Figure 8B) fibroblast cell culture supernatants treated with calcitriol and its analogs. The tested compounds resulted in a significant increase in the production of CCL2 by macrophages after 72 h of stimulation (*p* < 0.05, Figure 8A). Moreover, vitamin D compounds stimulated the secretion of OPN by fibroblasts after 24 h and 72 h, and OPN level was significantly higher in the supernatant of treated cells after 72 h of stimulation (*p* < 0.05, Figure 8B). There was no effect of calcitriol and its analogs on OPN secretion by macrophages (Figure 8A) and CCL2 secretion by fibroblasts (Figure 8B).

## 3. Discussion

The 4T1 murine mammary gland carcinoma is characterized by high metastatic potential and is considered as an animal model for stage IV human basal breast cancer [37,38]. Moreover, the growth of primary tumors developed by 4T1 cells is accompanied by strong leukocytosis and splenomegaly [39]. In our previous experiments, we showed that calcitriol and its analogs stimulated the metastasis of 4T1 cells in young mice [9]. However, in aged OVX mice, the opposite effect was seen, namely the transient decrease of the metastatic potential on day 28 of the experiment [36]. During 4T1 cancer progression, there was a systematic increase in the number of monocytes independently of the age of mice (Figure 1) [34]. Furthermore, on day 28, the administered compounds elevated the percentage of monocytes in young mice [34], while in aged OVX mice a decreasing trend was observed (Figure 1). Thus, an increase in the number of monocytes in the treated groups can be attributed to the higher metastatic potential in the young mice, whereas in the aged OVX mice the opposite trend was noticed. Wen et al. showed that increased value of absolute monocyte count is an independent prognostic value for shorter overall survival in breast cancer [40].

Two major subpopulations of monocytes can be distinguished in mice: classical Ly6C^high^CX3CR1^low^CCR2+ (referred to as inflammatory monocytes) and nonclassical “patrolling” Ly6C^low^CX3CR1^high^ (monocytes with repair and anti-inflammatory properties) [41]. In our study, the tested compounds significantly increased the ratio of Ly6C^low^:Ly6C^high^ monocytes in the splenocytes of young mice in an advanced stage of cancer progression, which was accompanied by an elevated number of lung metastases. Conversely, in the splenocytes of aged OVX mice with transient metastatic inhibition, the ratio of Ly6C^low^:Ly6C^high^ monocytes decreased in groups administrated with calcitriol and its analogs on the 28th day of the experiment. However, we did not notice similar dependence in the analysis of peripheral blood monocytes, both in the young and aged OVX mice (See: Supplementary Figure S1). It is postulated that stromal infiltration of tumor-associated macrophages (TAMs) originated from monocytes differentiation is a poor prognosis in various types of malignant tumors, including breast cancer. Moreover, it is underlined that the TAMs polarization status is also important, with an indication for an unfavorable prognosis when macrophages of M2 class are identified in the leukocyte infiltration (higher tumor growth, rapid progression, higher grade in the assessment of TNM, high Ki-67 proliferation index, and no expression of ER and PgR receptors) [42,43,44]. It was shown that the value of absolute peripheral monocyte count reflected the density of tumor infiltration by TAMs and was a predictive factor for prognosis and tumor progression in prostate [45] and colorectal cancers [46]. However, no unambiguous relationship between monocyte subpopulation type and polarization of the macrophage phenotype has yet been proven. Some scientific studies indicate that after infiltration into the tumor tissue, Ly6C^high^ monocytes can differentiate into both antitumor M1 and pro-tumor M2 macrophages [46,47]; however, other studies report that M1 macrophages are produced preferentially from Ly6C^high^ monocytes, while M2 macrophages are produced from Ly6C^low^ monocytes [48,49].

Our cytometric analysis of CD86 (M1 macrophage marker) and CD36 (M2 macrophage marker) surface markers of peritoneal macrophages obtained from young mice did not show the influence of calcitriol and its analogs on the phenotypic polarization of these cells (See: Supplementary Figure S2). In addition, the measurement of the cytokines level in the supernatants of peritoneal macrophages also did not allow for an unambiguous determination of the polarization status of macrophages (See: Supplementary Figure S3A). From the other hand, as we presented previously, calcitriol and its analogs elevated the levels of IL-10 in tumors of young mice [34], while in aged OVX mice the concentration of IL-10 level was not affected with a downward trend for PRI-2191 (current work, data not statistically significant). Additionally, in our present work, we have shown for young mice that in the late stage of cancer progression, PRI-2205 decreased the tumor level of iNOS (M1 macrophage marker) and a similar trend (data not statistically significant) was visible for PRI-2191. On the other hand, calcitriol stimulated the expression of Arg1 (M2 macrophage marker) in the tumor tissue of young mice bearing 4T1 cells. Interestingly, the opposite trend was noted for the aged OVX mice. It is postulated that IL-10 in the tumor microenvironment contributes to the silencing of immune response and polarization of macrophages to M2 class, with a suppressor and pro-tumoral properties [50,51]. Thus, our results may indicate that in the group of young mice treated with vitamin D compounds, immunosuppression of the tumor microenvironment could be intensified (in comparison to control mice), which could subsequently increase the metastatic potential of the tumor cells. Furthermore, macrophage colony-stimulating factor (M-CSF, encoded by *Csf1*) is an essential factor for the maturation of monocytes/macrophages. Ding et al. demonstrated that the more aggressive human MDA-MB-231 and MDA-MB-468 breast cancer cell lines show a higher expression of M-CSF than MCF-7 cells with less invasive phenotype [52]. Moreover, it has been noted that the enhanced expression of *Csf1* was correlated with a worse prognosis of overall survival in breast cancer [53] and with increased infiltration of TAMs in colorectal cancer [54]. Our real-time PCR analysis showed that in the lung tissue of young mice administered with calcitriol and its analogs, *Csf1* gene expression was found to be elevated in the late stage of tumor progression, whereas in aged OVX mice it decreased on 28th day in comparison to control mice, which correlated with metastatic potential. Moreover, the expression of *Plaur* encoding urokinase plasminogen activator receptor (uPAR) in lung tissue of aged OVX mice was also reduced after treatment with vitamin D compounds, while it was not affected in young mice. uPAR expression has a prognostic value in various malignancies, including breast cancer, and is involved in stimulating proliferation, migration and cellular invasion, degradation of the extracellular matrix, angiogenesis, and recruitment of macrophages [55,56].

Additionally, calcitriol and PRI-2191 increased the concentration of CCL2 chemokine in plasma and tumor tissues of young mice, while this effect was not observed in aged OVX mice. Chemokine CCL2 has strong chemotactic properties for classical monocytes, leading to their increased migration and infiltration to the site of inflammation. It is also believed that CCL2 is responsible for the polarization of the immune response toward Th2 cells and switching the macrophage phenotype to the M2 class [57]. It has been shown that CCL2 is overexpressed in breast cancer cell lines characterized as triple-negative [58], and silencing its expression led to inhibition of tumor progression depending on blocking the renewal of cancer stem cells and M2 macrophage polarization [59]. In studies conducted in the 4T1 model of breast carcinoma, Li et al. noted that anti-CCL2 therapy with blocking antibody led to a significant reduction in tumor volume and lung metastasis compared to the control group [60]. CCL2 expression is closely correlated with OPN expression and silencing of the *Spp1* gene led to significant inhibition of *Ccl2* gene expression. Moreover, OPN is a key regulator of the CCL2/CCR2 axis and by enhancing the signal transmission within the paths regulated by this chemokine, it contributes to the stimulation of the metastatic process [61]. OPN plays an important role in the metastasis of 4T1 cells, and it also participates in the formation of a metastatic niche [62,63]. In our previous studies conducted on young mice, we showed that increased metastasis in calcitriol and its analog-treated groups were accompanied by increased OPN expression in tumor [9] and lungs [34]. In contrast, in aged OVX mice, the level of OPN in the tumor was reduced [36]. Furthermore, an upward trend in the OPN concentration in plasma (difference was not statistically significant) of young mice [9] and a decrease of OPN concentration in aged OVX mice were observed [36]. Presented in current study analysis of the mRNA level of OPN in aged OVX mice also indicates that in groups treated with the tested compounds, there was a decrease in the expression of *Spp1*, especially in the lung tissue.

As we have found in our previous studies, the observed increase of OPN level in the tumor was not associated with its production by cancer cells themselves, as there was no elevated secretion of OPN in vitro by 4T1 cells after incubation with calcitriol and its analogs [9]. Therefore, we hypothesized that an increased level of OPN was a result of its excessive production by stromal cells present in the tumor microenvironment. To test this hypothesis, we estimated the concentration of OPN and CCL2 in mouse macrophage (RAW 264.7) and mouse fibroblast (BALB/3T3) cultures stimulated with the tested compounds. The effect of calcitriol on the proliferation of BALB/3T3 fibroblasts has been previously investigated [64]. Regarding RAW 264.7 macrophages, no cytotoxic effect of calcitriol was noted in the current work (See: Supplementary Figure S4). However, calcitriol and its analogs significantly increased OPN secretion in fibroblast cultures, whereas CCL2 production was increased only in macrophage cultures (Figure 8). This finding indicates that the tested compounds may have significant potential to modify the properties of stromal cells whose presence is found to be associated with cancer progression.

Moreover, calcitriol and PRI-2205 elevated the mRNA level of *Mmp14* in the lungs of young mice with increased metastatic nodules (late stage), while in aged OVX mice, reduced metastasis was demonstrated and all the compounds decreased the level of *Mmp14* expression (late-stage). In studies conducted on mouse mammary gland cancer cell lines 4T1 and E0771, it was proved that anti-MMP14 antibody administration inhibited tumor growth in both models [65]. Furthermore, it was noted that the applied therapy led to increased infiltration of macrophages with a switch to M1 phenotype (characterized by elevated expression of iNOS and decreased expression of CD206) and reduced signaling in TGF-β-regulated pathways [65]. In our studies, iNOS levels decreased in the tumor tissue of analog-treated young mice, and the TGF-β level was also found to be reduced in the tumors of these mice. On the other hand, TGF-β concentration in plasma of young mice was increased after treatment with vitamin D compounds in the late stage of tumor progression [9]; similarly, the level of *Tgfb1* mRNA in lung tissue increased in these mice (late-stage) [34]. In contrast, there was no effect of the tested vitamin D agents on TGF-β in tumor and plasma of aged OVX mice, while there was a significant decrease of *Tgfb1* expression in the lungs in treatment groups on day 28 of the experiment when the reduction of metastasis was evident. TGF-β is known for its immunosuppressive effect on the tumor stroma, participates in the formation of the metastatic niche, and could promote the formation of tumor blood vessels at low concentrations [66,67,68]. We have shown that in young mice, the tested compounds increased tumor blood perfusion [9], whereas in aged OVX mice this effect was not visible [36]. Increased tumor blood perfusion with lowering TGF-β level in young mice may indicate the normalization of tumor vascularity, which contributed to more efficient intravasation of cancer cells into the bloodstream. In addition, in the lung tissue of aged OVX mice vitamin D analogs decreased the expression of *Flt1* gene encoding vascular endothelial growth factor receptor 1 (VEGFR) in the late stage of tumor progression, whereas in young mice this effect was visible in the early stage and disappeared on 28th day of the experiment. Murakami et al. proved that VEGF contributes to increased vascular endothelial permeability [69], while Liu et al. in the studies conducted in the 4T1 model showed that VEGF plays a significant role in the formation of a metastatic niche in the lungs [70]. Therefore, it can be assumed that reduced VEGFR gene expression, observed in current work, in the aged OVX mice treated with vitamin D compounds may have contributed to worse rearrangement in the lung microenvironment, which might have resulted in a reduced number of metastases in these groups. In addition, in the lungs of aged OVX mice, there was a decrease of *Nedd9* expression in the late stage of tumor progression, whereas in young mice the expression of this gene was not affected. In studies conducted on bone marrow-derived macrophages and human dermal fibroblasts, it has been demonstrated that TGF-β may affect the transcription of NEDD9 [71]. It was shown that increased expression of NEDD9 was associated with a worse prognosis in breast cancer [72] and promotes cell invasion in aggressive breast cancer [73]. Moreover, this protein is a positive regulator of EMT, in which TGF-β also plays an important role. Thus, it appears that decreased expression of *Tgfb1* in aged OVX mice could affect the reduced level of *Nedd9* mRNA, which correlated with the lower number of metastases in these mice.

On the other hand, expression of tumor suppressor *Drg1* and *Nf2* was found to be elevated or decreased, respectively in the lungs of treated young or aged OVX mice at an advanced stage of cancer progression. However, it seems that an increase in the expression of *Spp1* and other described genes related to invasion and metastasis in young mice and decline of expression in aged OVX mice could outweigh the changes noted for *Nf2* and *Drg1* genes. Importantly, it should be noted that the expression profile of the studied genes in lung tissue, especially in the late stage of the experiment, is closely related to the metastatic cancer cells originated from the primary tumor. In fact, the analysis of the inflammatory markers *Ccr6*, *Cxcr4,* and *Foxo1* generally showed an increase in their expression only in the initial stage of tumor progression (Figure 3) when micrometastases begin to be present in the lungs [74], which can be explained as the initial mobilization of the immune system in response to a presence of metastatic cells. Interestingly, the activation of inflammation in young mice treated with PRI-2191 and PRI-2205 analogs is *Cxcr4*-dependent, while in the control and calcitriol-treated groups increased expression was noted for *Ccr6* and *Foxo1*. However, this seems to be irrelevant to the metastasis process itself, since in the later stage of cancer progression, when metastases are macroscopically present, differences in the expression of inflammation-related genes were not apparent, and also differences between young and aged OVX mice were not noted. Additionally, the differential effect of the PRI-2205 analog in relation to calcitriol or PRI-2191, observed in some analyses (e.g., CCL2 measurement), may result from a slightly different metabolism pathway of PRI-2205 (considered and explained in [75,76]).

**Figure 9:**
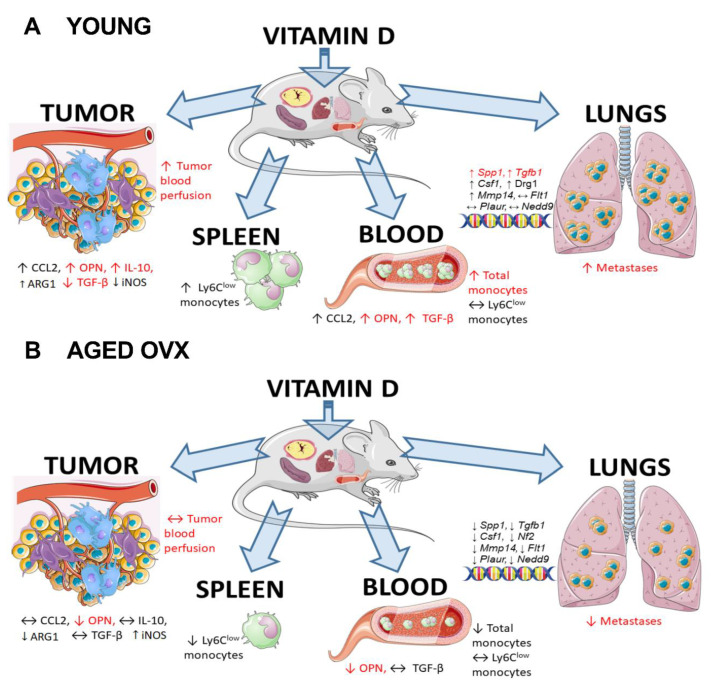
Vitamin D compounds effect on cancer progression in 4T1 mammary gland cancer model. Summary of results obtained for (**A**) young and (**B**) old OVX mice. In the case of young mice, in the groups receiving calcitriol and its analogs increased metastasis was observed, which was accompanied by upregulation in the expression of *Spp1*, *Tgfb1*, *Csf1*, *Drg1*, and *Mmp14* genes in the lung tissue. In these groups, elevated tumor blood perfusion and increased levels of CCL2, ARG1, OPN, and IL-10, with a simultaneous decrease of TGF-β and iNOS were noted. There was an increase in CCL2, OPN, and TGF-β levels in the blood accompanied by an elevated percentage of total monocytes. In the spleen, an increase in the percentage of Ly6C^low^ monocytes was noted in treatment groups, which was not visible in the blood. On the contrary, in old OVX mice, there was a decrease in the number of lung metastases and downregulation of *Spp1, Tgfb1, Csf1, Nf2, Mmp14, Flt1, Plaur,* and *Nedd9* gene expression in the lung tissue of treatment groups. Vitamin D compounds did not affect the tumor blood perfusion and levels of CCL2, IL-10, and TGF-β, while OPN and ARG1 expression in the tumor was reduced. A decrease in OPN and lack of changes in TGF-β levels were also observed in the blood in these groups, which was accompanied by a decrease in the percentage of total monocytes. In the spleen, a reduction of the percentage of Ly6C^low^ monocytes was noted, while in the blood there was no effect of vitamin D compounds on this monocyte subpopulation. In the figure, the **black** font indicates the results presented in the current work, while **red** font indicates our results described previously [9,34,36].

It is generally indicated that aging increases the risk of cancer, which is associated, among others, with a defective immune response and chronic inflammation caused by excessive production of pro-inflammatory cytokines by aging memory T lymphocytes and terminally differentiated TCD8+ cells [77]. Although the frequency of breast cancer increases with age, cases reported in young women are usually characterized by greater aggressiveness (basal and triple-negative breast cancer is more often diagnosed) and worse prognosis [78,79]. Moreover, it has been indicated that the reactivity and functionality of the immune system decrease in the elderly organism, and this condition is referred to as immunosenescence. It was shown that macrophages obtained from old mice were characterized by a smaller amount of secreted pro-inflammatory cytokines, lower MHC class II expression, and lower ability to present antigen and undergo phagocytosis [80]. In our experiments, the splenocytes of healthy aged OVX mice showed a significantly higher percentage of immunosuppressive Ly6C^low^ monocytes compared to young mice (Figure 2), which may indicate a lower basal reactivity of the immune system. On day 14, for both young and old mice, we saw a slight increase in Ly6C^high^ pro-inflammatory monocytes. In the following days, the ratio of Ly6C^low^:Ly6C^high^ for aged mice was similar to that on day 14, while in the young mice, we noted a progressive immunosuppression as manifested by a constant increase in the Ly6C^low^ population. In addition, Manhub et al. showed that when macrophages of aged mice were exposed to the cytokines necessary for determining the polarization type (M1 and M2), they differentiated less efficiently to individual classes than control macrophages of young mice, which indicates their functional disability in old age [81]. As mentioned previously, significant leukocytosis is observed in the 4T1 model. Thus, in the case of young mice with a properly functioning immune system, administration of calcitriol and its analogs could have triggered excessive immunosuppression of stromal cells infiltrating the tumor and might be involved in creating a metastatic niche, which subsequently increased the unfavorable pro-metastatic potential of cancer cells. In contrast, in the postmenopausal model, where the immune system is not as reactive and efficient as in the young organism, a negative influence of the tested compounds was not evident. A summary of the results is presented in Figure 9.

## 4. Materials and Methods

### 4.1. Compounds

Calcitriol (1,25-dihydroxyvitamin D_3_) and its two analogs, hereinafter referred to as PRI-2191 (tacalcitol, 1,24-dihydroxyvitamin D_3_) and PRI-2205 (5,6-trans-isomer of calcipotriol), were acquired from the Pharmaceutical Research Institute (Warsaw, Poland). Samples of the tested compounds were stored in amber ampoules in an atmosphere of argon at −20 °C. Immediately before use, the compounds were dissolved in 99.8% of ethanol (Avantor Performance Materials Poland, Gliwice, Poland) to obtain a final concentration of 10^−4^ M. Next, to achieve the required concentration, calcitriol and its analogs were diluted in an appropriate culture medium for in vitro studies and in 80% polyethylene glycol (Sigma-Aldrich, Saint-Louis, MO, USA) for in vivo studies.

Lipopolysaccharide (LPS) from *Escherichia coli* 0111:B4 (Sigma-Aldrich, Saint-Louis, MO, USA) was dissolved in phosphate-buffered saline (PBS) solution (IIET, PAS, Wroclaw, Poland) at a concentration of 1 mg/mL and stored at −20 °C. Immediately before use, LPS samples were dissolved in the appropriate culture medium to obtain a working concentration of 1 µg/mL for ex vivo studies.

### 4.2. Cells and Cell Line Cultures

Mouse mammary adenocarcinoma 4T1 cell line was purchased from American Type Culture Collection (ATCC, Rockville, MD, USA). The 4T1 cells were cultured in a mixture of RPMI-1640 and Opti-MEM medium (1:1; Thermo Fisher Scientific, Waltham, MA, USA) with an addition of 3.5 g/L glucose, 2 mM l-glutamine, 0.5 mM sodium pyruvate (Sigma-Aldrich, Saint-Louis, MO, USA), and 5% fetal bovine serum (FBS, HyClone; Thermo Fisher Scientific, Waltham, MA, USA). Murine macrophage-like cell line RAW 264.7 was obtained from Leibniz Institute DSMZ-German Collection of Microorganisms and Cell Cultures (Braunschweig, Germany) and normal mouse fibroblast cell line BALB/3T3 was purchased from ATCC (Rockville, MD, USA). Both cell lines were maintained in Dulbecco’s modified Eagle’s medium (Thermo Fisher Scientific, Waltham, MA, USA) containing 10% FBS (HyClone; Thermo Fisher Scientific, Waltham, MA, USA) and 2.0 mM l-glutamine (Sigma-Aldrich, Saint-Louis, MO, USA). All the culture media were supplemented with 100 U/mL of penicillin (Polfa Tarchomin S.A., Warsaw, Poland) and 100 µg/mL of streptomycin (Sigma-Aldrich, Saint-Louis, MO, USA). The 4T1, BALB/3T3, and RAW 264.7 cells were cultured at 37 °C in a humidified atmosphere with 5% CO_2_.

### 4.3. Mouse Studies

We collected blood, peritoneal fluid, spleen, lungs, and tumor tissue from BALB/c female mice inoculated orthotopically with 4T1 mammary gland cancer cells (1 × 10^4^ viable cells per mice). For ethical reasons, we decided to use the biological material collected from mice during our previous studies, without the need to repeat the whole experiment. The scheme of conducting the experiment has been described in [9,37]. All the experiments involving animals have been carried out according to the European Union (EU) Directive 2010/63/EU and have been approved by the First Local Committee for Experiments with the Use of Laboratory Animals, Wroclaw, Poland (No. of permission 40/2014, 16 July 2014).

Briefly, young (6–8-week-old) mice weighing 20–25 g and aged (60-week-old) mice weighing 24–34 g were purchased from the Center of Experimental Medicine of the Medical University of Bialystok (Bialystok, Poland). In the case of aged mice, ovariectomy was performed to obtain a postmenopausal model, including mice subjected to the same surgical procedure without ovariectomy served as an additional control (Sham). Four weeks after the procedure, the actual experimental protocol was started. Both young and aged mice were subjected to the same experimental procedure. The day on which 4T1 cells were inoculated was treated as day 0. Next, subcutaneous administration of calcitriol (0.5 µg/kg) and its analogs—PRI-2191 (1 µg/kg) and PRI-2205 (10 µg/kg)—was started 7 days after transplantation of cancer cells and was conducted three times a week. Polyethylene glycol (80%), a solvent for vitamin D compounds, was used as a vehicle control. On days 14–21 (early stage of tumor progression) and 28–33 (late stage of tumor progression), the mice were euthanized to collect the biological material for further analysis.

### 4.4. Blood Preparation

Blood was collected immediately after performing the euthanasia procedure. The level of monocytes in the whole blood sample was determined using a hematology analyzer Mythic 18 (Cormay, Lomianki, Poland). Then, the blood specimens were centrifuged at 2000× *g* for 15 min at 4 °C. After this step, plasma was transferred to fresh tubes and frozen at −20 °C for further analysis by ELISA. Morphotic elements were resuspended in HBSS buffer (IIET PAS, Wroclaw, Poland) and layered on a Ficoll-Paque 1.084 solution (Sigma-Aldrich, Saint-Louis, MO, USA). After centrifugation at 400× *g* for 45 min at room temperature, a buffy coat containing mononuclear cell fraction (PBMCs) was collected and washed twice by using PBS supplemented with 2% FBS. The obtained cells were then subjected to cytometric analysis.

### 4.5. Peritoneal Fluid Preparation

The peritoneal cavity was injected with 5 mL of cold PBS with 2% FBS. Then, peritoneal fluid was harvested and transferred to a sterile tube. Peritoneal fluid was washed twice with PBS + 2% FBS. Next, the cell suspension was seeded on a Petri dish for 2 h in RPMI 1640 with 10% FBS, 2.0 mM l-glutamine, and antibiotics. Later, nonadherent cells were washed out by rinsing with PBS. Peritoneal macrophages were harvested from dishes by using cell scrapers (BD Biosciences, San Jose, CA, USA) and subjected to cytometric analysis.

Simultaneously, 1 × 10^6^ peritoneal macrophages in 1 mL of RPMI 1640 medium supplemented with 10% FBS, 2.0 mM l-glutamine, and antibiotics were seeded on a 24-well plate (Corning, New York, NY, USA). To stimulate cytokine production, fresh medium containing 1 µg/mL of LPS solution was added after 24 h. After incubating further for 24 h, cell culture supernatant was collected, centrifuged, and frozen at −20 °C for quantification by ELISA.

### 4.6. Spleen Tissue Preparation

The spleens were collected in RPMI 1640 medium containing 2% FBS. Subsequently, the tissue was mechanically crushed, and a single-cell suspension was obtained by passing through a cell strainer with a pore diameter of 70 µm. The obtained splenocytes were then subjected to cytometric analysis.

### 4.7. Lung Tissue Preparation

Lung samples were transferred into tubes contained TRI reagent (Sigma-Aldrich, Saint-Louis, MO, USA), and RNA was isolated according to the standard protocol provided by the manufacturer. Then, the quantity and purity of RNA were determined spectrophotometrically at 260 nm by using NanoDrop 2000 Spectrophotometer (Thermo Fisher Scientific, Waltham, MA, USA). 1 µg of RNA samples were purified from the genomic DNA with 1 U per sample of DNase I (Thermo Fisher Scientific, Waltham, MA, USA) in the presence of RNase inhibitors (50 U per sample; EURx, Gdansk, Poland). cDNA was obtained using iScript cDNA Synthesis Kit (Bio-Rad, Hercules, CA, USA)—1 µg of purified RNA was mixed with 1 µL of iScript reverse transcriptase and 4 µL of iScript reaction mix and the following protocol was used: priming (5 min at 25 °C), reverse transcription (20 min at 46 °C) and RT inactivation (1 min at 95 °C). The genetic material obtained in this way was used to perform the quantitative polymerase chain reaction (PCR) to estimate the expression of specific genes.

### 4.8. Tumor Tissue Preparation

RNA samples, for analysis by quantitative PCR, were prepared from the tumor tissue as described in the previous section. Additionally, tumor specimens were frozen in liquid nitrogen, and then crushed and resuspended in RIPA buffer containing protease inhibitors (both Sigma-Aldrich, Saint-Louis, MO, USA). After mechanical homogenization (MP Biomedicals, Santa Ana, CA, USA), samples were centrifuged at 14,000× *g* for 10 min at 4 °C and supernatants were transferred into fresh tubes to determine the concentration of protein (DC Protein assay; Bio-Rad, Hercules, CA, USA). The protein samples obtained from tumor tissue were further used for ELISA assays.

### 4.9. Flow Cytometry Analysis

Peripheral blood mononuclear cells, splenocytes, or peritoneal macrophages at a concentration of 1 × 10^6^ cells were centrifuged at 192× *g* for 7 min at 4 °C. Subsequently, samples were incubated with anti-CD16/CD32 (BD Biosciences, San Jose, CA, USA) antibodies for 10 min to block the Fc receptors. After this, PBMCs and splenocytes were incubated with the rat anti-mouse antibodies CD11b-APC, Ly-6C-PE, and Ly-6G-FITC (BD Biosciences, San Jose, CA, USA), while peritoneal macrophages were stained with rat anti-mouse antibodies CD45-PerCP-Cy5.5, CD11b-APC, CD36-PE, CD86-FITC, and Ly6G/Ly6C-APC-Cy7 (BD Biosciences, San Jose, CA, USA) for 30 min at 4 °C in the dark. Subsequently, cells were centrifuged and resuspended in PBS supplemented with 2% FBS. Samples were analyzed in BD LSR Fortessa cytometer with FACSDiva v8.0.1 software (BD Biosciences, San Jose, CA, USA).

### 4.10. Real-Time PCR Analysis

The PCR reactions were performed based on Taq-Man chemistry using the probes specific for the following genes: *Csf1* (Mm00432686_m1), *Ccr6* (Mm99999114_s1), *Cxcr4* (Mm01996749_s1), *Drg1* (Mm00492246_m1), *Foxo1* (Mm00490672_m1), *Nf2* (Mm00477771_m1), *Nedd9* (Mm01324843_m1), *Mmp14* (Mm00485054_m1), *Spp1* (Mm00436767_m1), *Flt1* (Mm00438980_m1), *Plaur* (Mm01149438_m1), *Tgfb1* (Mm01178820_m1), *Pgk1* (Mm00435617_m1), and *Rpl13a* (Mm01612987_g1) (all Thermo Fisher Scientific, Waltham, MA, USA). Each amplification cycle was performed at 95 °C for 15 s and at 60 °C for 1 min (total 40 cycles) in ViiA™ 7 Real-Time PCR System (Thermo Fisher Scientific, Waltham, MA, USA). Twenty-five nanograms of cDNA were used for a single reaction and each sample was prepared in triplets (technical repetition). The relative quantification level of examined gene expression, referred to as fold change, was calculated based on differences in ΔΔCt values of the studied genes in relation to control housekeeping genes (*Pgk1* or *Rpl13a*) by using DataAssist 3.01 software (Thermo Fisher Scientific, Waltham, MA, USA).

### 4.11. ELISA Assay

To assess the expression of selected proteins in mouse plasma and tumor homogenates, ELISA kits were used, and the assay was performed according to the manufacturer’s instructions. Absorbance was measured immediately after completion of the protocol by plate reader Synergy H4 (BioTek Instruments, Winooski, VT, USA) at the wavelength indicated by the manufacturer. Expression of the following cytokines and proteins was examined: iNOS, Arg1 (both EIAab, Wuhan, China), OPN (R&D Systems, Minneapolis, MN, USA), IL-1β, IL-6, IL-10, CCL2, and TGF-β (all Thermo Fisher Scientific, Waltham, MA, USA).

### 4.12. In Vitro Studies

Cytotoxicity assay for RAW 264.7 murine macrophages was performed as previously described [75]. In short, 1.5 × 10^3^ cells per well in 100 µL of appropriate medium were seeded on a 96-well plate (Sarstedt, Nümbrecht, Germany). The following day, vitamin D supplements were added at concentrations of 1–1000 nM in triplicates (technical repetition) and incubated for 72 h. After this period, the SRB assay protocol was performed. Absorbance was measured immediately after staining by plate reader Synergy H4 at a wavelength of 540 nm. Four independent repetitions were conducted. In addition, control of vitamin D compounds solvent was performed (99.8% ethanol) and the results were compared to those obtained for the reference compound, cisplatin. The rate of inhibition of proliferation of the treated cells in relation to the untreated cells was calculated. IC_50_ values—the dose that inhibits proliferation of 50% of macrophages—were calculated in Prolab-3 system based on Cheburator 0.4, Dmitry Nevozhay software [82].

Additionally, to determine the expression of the selected proteins, ELISA assay was performed on BALB/3T3/RAW 264.7 cell culture supernatants. Briefly, 3 × 10^5^ cells were seeded on a sterile Petri dish in a suitable medium. The following day, calcitriol and its analogs were added at a concentration of 100 nM, and cell cultures were incubated for 24 or 72 h. After this period, supernatants were collected, centrifuged, and frozen at −20 °C for quantification of CCL2 and OPN.

### 4.13. Statistical Evaluation

Statistical analysis was conducted using GraphPad Prism 7.01 (GraphPad Software Inc., San Diego, CA, USA). Shapiro–Wilk’s normality test and Bartlett’s test were performed to check the assumptions for analysis of variance (ANOVA). Tests used for each data analysis are indicated in the figure legends. *p*-value < 0.05 was considered significant.

## 5. Conclusions

In our experiments, we showed that calcitriol and its analogs affect cancer progression in a different manner (pro-metastatic or anti-metastatic action), depending on the age of the organism being studied. This study suggests that in the model of young mice calcitriol and its derivatives, owing to their anti-inflammatory properties, can enhance the immunosuppression of the tumor microenvironment; however, this effect was not seen in aged OVX mice, and presumably, this may be the result of differences in the immunity status of the young and old organisms. We were the first to show that vitamin D may exert an unfavorable effect on the progression of breast cancer under specific conditions. We indirectly proved that the reason for the occurrence of such a phenomenon was the suppressive stimulation of the tumor microenvironment by vitamin D compounds. However, investigations should be continued to determine the exact mechanism of this influence, which could increase the knowledge about the safety of vitamin D supplementation in people with advanced stages of invasive tumors.

Moreover, the results of our study have demonstrated that the activity of the tested compounds in a specific cancer model may differ radically depending on the age of the mouse. Due to the fact that preclinical studies in the field of experimental oncology are most often carried out on young mice, we point out the need to conduct the experiments in both young and elderly organisms.

## Supplementary Materials

Supplementary materials can be found at https://www.mdpi.com/1422-0067/21/17/6359/s1.

## Abbreviations

ARG1	arginase 1
CCL2	chemokine (C-C motif) ligand 2
CXCL1	chemokine (C-X-C motif) ligand 1
iNOS	inducible nitric oxide synthase
MCS-F	macrophage colony-stimulating factor
OPN	osteopontin
OVX	ovariectomy
TAMs	tumor-associated macrophages
TGF-β	transforming growth factor be
TME	tumor microenvironment
TNF-α	tumor necrosis factor alpha
uPAR	urokinase plasminogen activator receptor
VDC	vitamin D compounds
VEGFR	vascular endothelial growth factor receptor 1
VDR	vitamin D receptor
