# Supplementary material for: Differential Impact of Calcitriol and Its Analogs on Tumor Stroma in Young and Aged Ovariectomized Mice Bearing 4T1 Mammary Gland Cancer
Source: Int J Mol Sci. 2020 Sep 2;21(17):6359. doi: 10.3390/ijms21176359 (PMC7503326; doi:10.3390/ijms21176359)
Supplement: Supplementary file 1 [file ijms-21-06359-s001.pdf]

Supplementary Results

Differential Impact of Calcitriol and Its Analogs on Tumor Stroma in Young and Aged Ovariectomized Mice Bearing 4T1 Mammary Gland Cancer

Artur Anisiewicz *, Agata Pawlik, Beata Filip-Psurska and Joanna Wietrzyk

Department of Experimental Oncology, Hirszfeld Institute of Immunology and Experimental Therapy, Polish Academy of Sciences, 53-114 Wroclaw, Poland; agata.maria.pawlik@gmail.com (A.P.); beata.filip-psurska@hirszfeld.pl (B.F.-P.); joanna.wietrzyk@hirszfeld.pl (J.W.)

* Correspondence: artur.anisiewicz@hirszfeld.pl

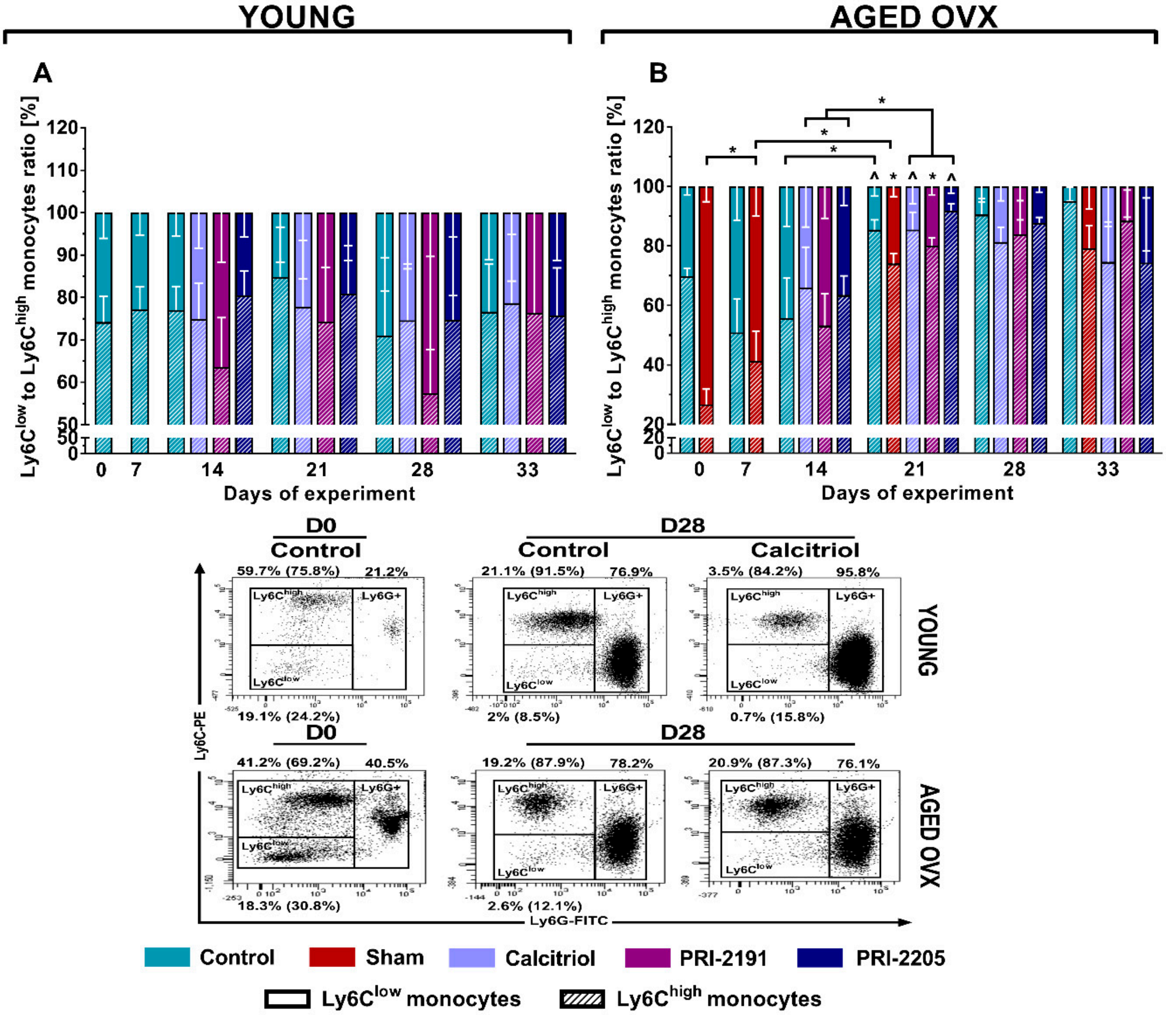

Supplementary Figure S1. The Ly6C^{low} to Ly6C^{high} ratio of whole blood monocytes in 4T1 tumor-bearing young and aged OVX mice. Ly6C^{low} to Ly6C^{high} ratio of whole blood monocytes in (A) young or (B) aged OVX mice. (C) Representative dot plots of selected analysis with the % of cells of each gate performed

on day 0 or 28. The values in parentheses represent the percentage of monocytes of individual classes among all monocytes. About 1 × 10^6 viable cells were used for cytometric analysis. Firstly, monocytes were gated as a CD11b-positive and Ly6G-negative cells (gating strategy according to Fig. 2D). Then monocytes were differentiated due to the expression of the Ly6C marker. Briefly, 60–week–old mice were subjected to ovariectomy or sham surgery. After 4 weeks, 4T1 cells were inoculated (day 0) orthotopically into aged OVX mice or 6–8-week-old young mice and subcutaneous administration of tested compounds (three times a week) was initiated on day 7 at the following doses: calcitriol, 0.5 µg/kg; PRI-2191, 1.0 µg/kg; PRI-2205, 10.0 µg/kg. The number of mice analyzed was 3–7 per group. Data presentation: (A) and (B), mean with standard deviation (SD). Statistical analysis: (A) Dunn's multiple comparison test and (B) Dunnett's multiple comparison test. *P<0.05 as compared to control animals or ^P<0.05 as compared do control sham animals on the relevant day of treatment or as indicated.

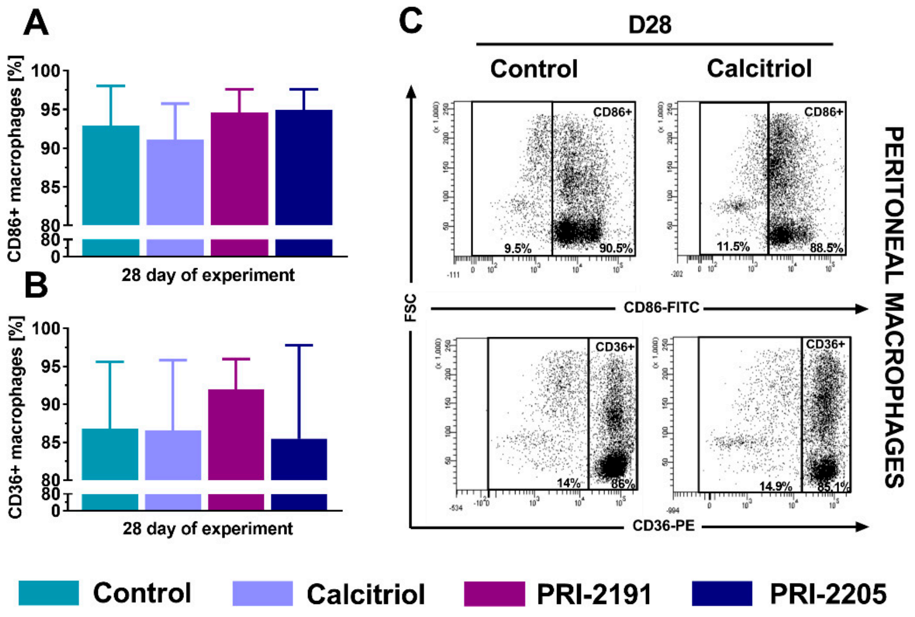

Supplementary Figure S2. Expression of CD86 and CD36 markers on peritoneal macrophages of 4T1 tumor-bearing young and aged OVX mice. (A) CD86 and (B) CD36 expression was determined in peritoneal macrophages of young mice on 28th day of the experiment. (C) Representative dot plots of selected analysis with the % of cells of each gate performed on day 28. About 1 × 10^6 viable cells were used for cytometric analysis. Firstly, macrophages were gated as a CD11b-and CD45–positive and Ly6C/Ly6G-negative cells. Then macrophages underwent differentiation due to the expression of CD86 and CD36 markers. Briefly, 4T1 cells were inoculated (day 0) orthotopically into 6–8-week-old young mice and subcutaneous administration of tested compounds (three times a week) was initiated on day 7 at the following doses: calcitriol, 0.5 µg/kg; PRI-2191, 1.0 µg/kg; and PRI-2205, 10.0 µg/kg. The number of mice analyzed was 5–6 per group. Data presentation: (A) and (B), mean with standard deviation (SD).

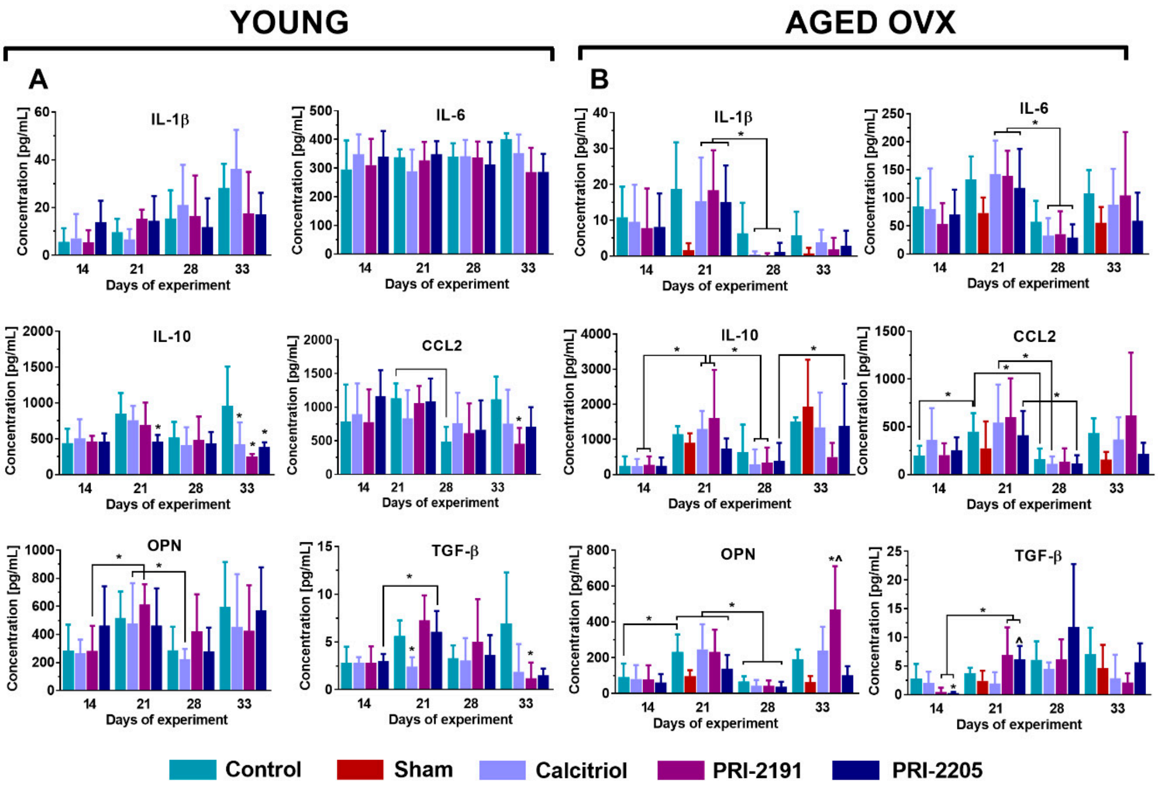

Supplementary Figure S3. Changes related to protein expression in supernatants collected from peritoneal macrophages obtained from 4T1 tumor-bearing young or aged OVX. Expression of selected proteins in peritoneal macrophage supernatants collected from (A) young or (B) aged OVX mice measured using ELISA assays. The analysis was performed on specimens harvested on days 14, 21, 28, and 33. Briefly, 60-week-old mice were subjected to ovariectomy or sham surgery. After 4 weeks, 4T1 cells were inoculated (day 0) orthotopically into aged OVX mice or 6–8-week-old young mice and subcutaneous administration of the tested compounds (three times a week) was initiated on day 7 at the following doses: calcitriol, 0.5 µg/kg; PRI-2191, 1.0 µg/kg; and PRI-2205, 10.0 µg/kg. The number of mice analyzed was 3–7 per group. Data presentation: (A) and (B), mean with standard deviation (SD). Statistical analysis: (A) and (B), Dunn's multiple comparison test. **P*<0.05 as compared to control animals or ^*P*<0.05 as compared do control sham animals on the relevant day of treatment or as indicated. IL-1β—interleukin 1β; IL-6—interleukin 6; IL-10—interleukin 10; CCL2—chemokine (C-C motif) ligand 2; OPN—osteopontin; TGF-β—transforming growth factor β.

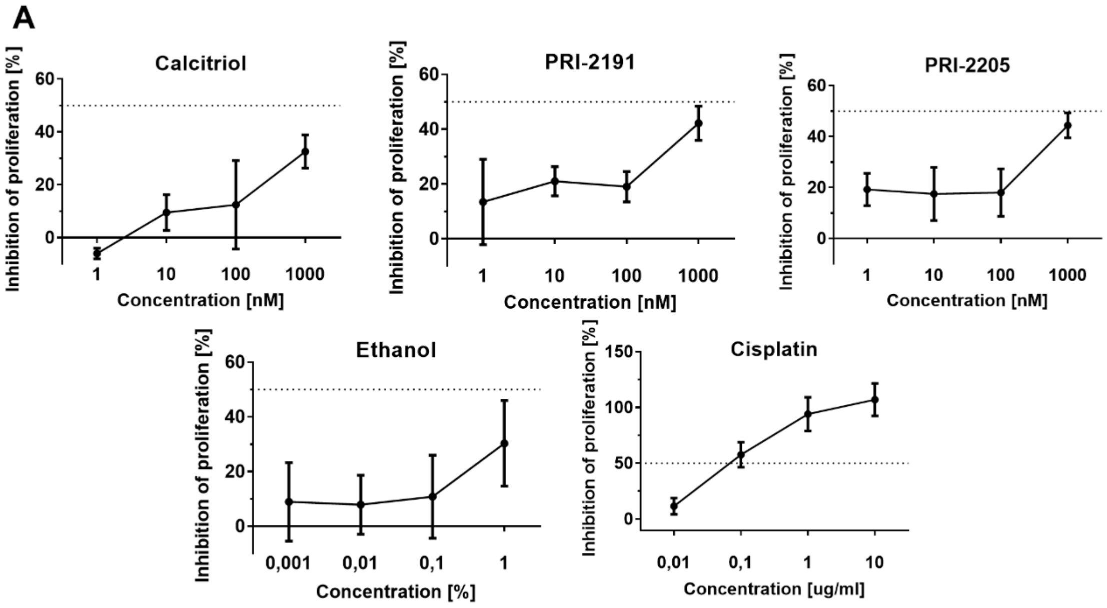

Supplementary Figure S4. Influence of calcitriol and its analogs on the proliferation of RAW 264.7 murine macrophages. About 1.5 x 10^3 cells per well were seeded on a 96-well plate. The following day, vitamin D compounds were added at concentrations in the range of 1–1000 nM in triplicates and incubated for 72 h, and four independent repetitions were performed. Then, SRB assay protocol was conducted. Absorbance was measured at a wavelength of 540 nm. In addition, control of vitamin D compounds solvent was performed (99.8% ethanol) and the results were compared to those obtained for the reference compound, that is, cisplatin. The rate of inhibition of proliferation of treated cells was calculated in relation to that of untreated cells. Data presentation: (A), mean with standard deviation (SD).
